# Humoral response to SARS-CoV-2 mRNA vaccination in previous non-responder kidney transplant recipients after short-term withdrawal of mycophenolic acid

**DOI:** 10.3389/fmed.2022.958293

**Published:** 2022-08-18

**Authors:** Louise Benning, Christian Morath, Tessa Kühn, Marie Bartenschlager, Heeyoung Kim, Jörg Beimler, Mirabel Buylaert, Christian Nusshag, Florian Kälble, Marvin Reineke, Maximilian Töllner, Matthias Schaier, Katrin Klein, Antje Blank, Paul Schnitzler, Martin Zeier, Caner Süsal, Ralf Bartenschlager, Thuong Hien Tran, Claudius Speer

**Affiliations:** ^1^Department of Nephrology, University of Heidelberg, Heidelberg, Germany; ^2^Department of Infectious Diseases, Molecular Virology, University of Heidelberg, Heidelberg, Germany; ^3^Department of Clinical Pharmacology and Pharmacoepidemiology, University of Heidelberg, Heidelberg, Germany; ^4^German Center for Infection Research, Partner Site Heidelberg, Heidelberg, Germany; ^5^Department of Virology, University of Heidelberg, Heidelberg, Germany; ^6^Transplant Immunology Research Center of Excellence, Koç University Hospital, Istanbul, Turkey; ^7^Division Virus-Associated Carcinogenesis, German Cancer Research Center, Heidelberg, Germany; ^8^Institute of Immunology, University of Heidelberg, Heidelberg, Germany; ^9^Department of Molecular Medicine Partnership Unit Heidelberg, European Molecular Biology Laboratory, Heidelberg, Germany

**Keywords:** SARS-CoV-2, kidney transplantation, variants of concern, delta variant, omicron variant, SARS-CoV-2 vaccination

## Abstract

Seroconversion rates after COVID-19 vaccination are significantly lower in kidney transplant recipients compared to healthy cohorts. Adaptive immunization strategies are needed to protect these patients from COVID-19. In this prospective observational cohort study, we enrolled 76 kidney transplant recipients with no seroresponse after at least three COVID-19 vaccinations to receive an additional mRNA-1273 vaccination (full dose, 100 μg). Mycophenolic acid was withdrawn in 43 selected patients 5–7 days prior to vaccination and remained paused for 4 additional weeks after vaccination. SARS-CoV-2-specific antibodies and neutralization of the delta and omicron variants were determined using a live-virus assay 4 weeks after vaccination. In patients with temporary mycophenolic acid withdrawal, donor-specific anti-HLA antibodies and donor-derived cell-free DNA were monitored before withdrawal and at follow-up. SARS-CoV-2 specific antibodies significantly increased in kidney transplant recipients after additional COVID-19 vaccination. The effect was most pronounced in individuals in whom mycophenolic acid was withdrawn during vaccination. Higher SARS-CoV-2 specific antibody titers were associated with better neutralization of SARS-CoV-2 delta and omicron variants. In patients with short-term withdrawal of mycophenolic acid, graft function and donor-derived cell-free DNA remained stable. No acute rejection episode occurred during short-term follow-up. However, resurgence of prior anti-HLA donor-specific antibodies was detected in 7 patients.

## Introduction

Kidney transplant recipients (KTR) are at high risk for severe COVID-19 infection with an overall reported 28-day probability of COVID-19 related death of 21.3% and a twofold higher risk of death in KTR compared to non-transplanted patients (1–3). Response to vaccination is significantly impaired in KTR compared to healthy cohorts even after three doses of an mRNA vaccine (4–13). Furthermore, vaccine-induced SARS-CoV-2 specific antibodies wane over time in KTR and healthy cohorts alike, facilitating breakthrough infections with higher viral load (14–16). With the surge of the highly transmissive immune-escaping B.1.1.529 (omicron) variant, KTR remain at risk for COVID-19 disease. A fourth vaccine dose has been recommended recently in several countries for the elderly and immunocompromised, however, seroconversion rates in KTR with low or no antibody response after three vaccine doses after an additional fourth vaccine dose remain low and range between 42 and 50% (17–21).

Neutralizing antibodies are considered a strong predictor of protection from symptomatic COVID-19 disease (22–26). We and others showed that lower anti-spike antibodies in KTR are concomitant with lower or even absent neutralization of variants of concern such as the B.1.617.2 (delta) or B.1.1.529 (omicron) variant (13, 27–29). Therefore, seropositivity in commercially available assays testing for antibodies to the wild-type spike antigen may result in an overestimation of actual protection against viral variants (13, 22, 27, 30).

To enhance vaccination responsiveness and to better protect KTR from COVID-19 disease, adaptive immunization strategies for KTR are urgently needed. One attempt to enhance seroconversion in KTR is through modulation of immunosuppression as especially patients treated with mycophenolic acid (MPA) have shown significantly impaired seroconversion rates when compared to KTR with other immunosuppressive maintenance regimens (31–33).

In this study, we aimed to determine the effect of an additional full elasomeran dose (100 μg), formerly known as mRNA-1273, in non-responder KTR with at least 3 previous vaccine doses of any COVID-19 vaccine. In KTR with triple immunosuppressive therapy including a calcineurin inhibitor (CNI), MPA and corticosteroids (CS), MPA was withdrawn in those with stable graft function and no prior rejection in the past 12 months to investigate the efficacy of short-term MPA withdrawal on COVID-19 vaccine immunogenicity.

## Materials and methods

### Study design

**FIGURE 1:**
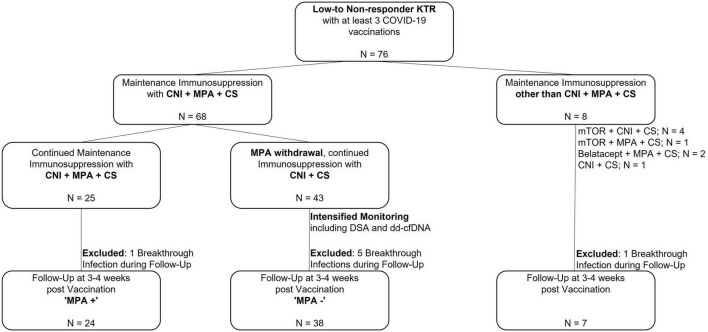
Study flow chart to assess humoral responses to an additional COVID-19 vaccination in 76 non-responder kidney transplant recipients after an additional mRNA-1273 vaccine dose. A total of 76 kidney transplant recipients (KTR) with different immunosuppressive regimens with no seroconversion after at least 3 COVID-19 vaccine doses were included in this trial. Short-term withdrawal of mycophenolic acid (MPA) during vaccination was evaluated in 68 KTR with maintenance immunosuppression consisting of a calcineurin inhibitor (CNI), MPA and corticosteroids (CS). In 25 KTR, triple immunosuppressive therapy was maintained (“MPA + “), whereas MPA was paused in 43 KTR (“MPA-”). These 43 KTR received intensified monitoring including testing for donor-specific HLA antibodies (DSA) and donor-derived cell-free DNA (dd-cfDNA) prior to and after withdrawal of MPA. In addition, humoral response was assessed in 8 KTR with immunosuppressive maintenance therapy other than CNI, MPA and CS. Breakthrough infections during the 4 weeks post vaccination surveillance period occurred in all three groups with 1 breakthrough infection in the group where maintenance immunosuppression with CNI, MPA and CS was maintained, 5 breakthrough infections in the group where MPA was withdrawn temporarily, and 1 breakthrough infection in the group with maintenance immunosuppression other than CNI, MPA and CS. Thus, follow-up for humoral response was reduced to 69 KTR. CNI, calcineurin inhibitor; CS, corticosteroids; KTR, kidney transplant recipients; MPA, mycophenolic acid; mTOR, mammalian target of rapamycin; N, number.

We enrolled 76 KTR with an anti-spike S1 IgG antibody index ≤ 10 after at least three COVID-19 vaccinations to participate in this prospective observational cohort study between January and February 2022 at the Department of Nephrology, University of Heidelberg, Germany. The cut-off > 10 was identified as we previously showed that an anti-spike S1 IgG antibody index > 10 significantly correlated with the presence of wild-type SARS-CoV-2 neutralizing antibodies (13, 14). An additional mRNA-1273 vaccine dose (full dose, 100 μg) was administered to the identified patients. Serum for analysis of humoral responses to vaccination was drawn immediately before and with a median (IQR) of 27 (27–30) days after vaccination. Patients with a history of prior SARS-CoV-2 infection and/or detectable anti-nucleocapsid antibodies were excluded from the study. Further, we excluded 7 patients with PCR-confirmed breakthrough infections during follow-up from analysis (Figure 1).

Patients were stratified according to current immunosuppressive maintenance therapy. Summarized, short-term withdrawal of MPA was discussed in patients with a triple immunosuppressive maintenance therapy (CNI, MPA, and CS) in case graft function was stable (defined as S-creatinine ≤ 2.5 mg/dl and proteinuria ≤ 2 g/l) and no graft rejection the past 12 months, an anti-spike S1 IgG antibody index ≤ 10 after at least three COVID-19 vaccinations and no prior SARS-CoV-2 infection. Decision on short-term withdrawal of MPA was based on shared decision-making after detailed information of the patient and performed according to our department’s standard operating procedure for MPA withdrawal upon infection/vaccination. In 43 patients, MPA was consecutively withdrawn 5-7 days prior to vaccination and remained paused for additional 4 weeks after vaccination (Figure 1). Donor-specific anti-HLA antibodies (DSA) and donor-derived cell-free DNA (dd-cfDNA) were determined in addition to routine transplant laboratory prior to MPA withdrawal and at follow-up. The formation of *de novo* DSA was evaluated including prior DSA testing available from post-transplant routine laboratory.

Humoral response to COVID-19 vaccination was assessed by determination of anti-spike S1 IgG, surrogate neutralizing, and anti-receptor-binding domain (anti-RBD) antibodies. In addition, IgG antibodies targeting the SARS-CoV-2 full spike, the spike S1 and S2 subunits, and the nucleocapsid protein were measured. Neutralization of the B.1.617.2 (delta) and the B.1.1.529 (omicron) variants of concern was determined in all KTR after additional COVID-19 vaccination using a live-virus assay.

The study was approved by the ethics committee of the University of Heidelberg and conducted in accordance with the Declaration of Helsinki. Written informed consent was obtained from all study participants. The study is registered at the German Clinical Trial Register (DRKS00024668).

### Assessment of humoral responses after COVID-19 vaccination with three commercially available tests

Anti-Spike S1 IgG and anti-nucleocapsid antibodies were determined by using the SARS-CoV-2 Total Assay (Siemens, Eschborn, Germany) and the Elecsys anti-SARS-CoV-2 assay (Roche, Mannheim, Germany), respectively. The anti-SARS-CoV-2 IgG spike assay was calibrated with two different calibrators containing low and high concentrations of the spike protein. After calibration, the system calculated an index as cut-off, values < 1.0 were reported as negative and values ≥ 1.0 were reported as positive according to the manufacturer’s instructions. An index value of 1 corresponds to 21.8 binding antibody units (BAU) per milliliter according to the World Health Organization’s international standard for anti-SARS-CoV-2 immunoglobulin (34, 35). Surrogate neutralizing antibodies were measured using a surrogate virus neutralization assay (Medac, Wedel, Germany). The assay mimics the virus-host interaction by direct protein-protein interaction using purified RBD from the viral spike and the ACE-2 host cell receptor (36). IgG antibodies against the SARS-CoV-2 full spike, the spike S1 and spike S2 subunits, and the RBD protein were detected using a bead-based multiplex assay for the Luminex platform (LabScreen Covid Plus, One Lambda, Inc., West Hill, CA, United States). This assay further determines IgG antibody reactivity against the spike S1 of four common cold coronaviruses (HCoV-229E, HCoV-HKU1, HCoV-NL63, and HCoV-OC43) (37). All assays have been described in previous works and were performed according to the manufacturer’s instructions (38–41).

### Live-virus neutralization against the B.1.617.2 (delta), and the B.1.1.529 (omicron) variant

Neutralization titers were determined in twofold serial dilution experiments using VeroE6 cells, as described previously (13, 14, 27, 42–47). Virus stocks were produced by isolation and amplification of the B.1.617.2 (delta) and the B.1.1.529 (omicron) variant from nasopharyngeal and oropharyngeal swabs of PCR-confirmed SARS-CoV-2 positive patients (27, 48). B.1.617.2 (delta) variant was amplified in VeroE6 cells. Stocks of B.1.1.529 (omicron) were produced in Calu-3 cells to avoid rapid cell culture adaptation. Virus titers of stocks were determined by plaque assay and Tissue Culture Infectious Dose (TCID) 50 assay in VeroE6 cells. To validate virus stocks, genome sequencing was performed. For the neutralization assays, twofold serial dilutions of vaccine sera were incubated with 6 × 104 TCID 50 of the B.1.617.2 (delta) and the B.1.1.529 (omicron) variant. Virus replication was determined by immunostaining for the viral nucleocapsid protein using an in-cell ELISA. Data were normalized to a no-serum (100%) and a mock-infected (0%) control. The serum dilution that results in 50% reduction of normalized signal gives the inhibitory dilution 50 (ID_50_).

### Determination of donor-specific anti-HLA antibodies (DSA)

In all patients in whom MPA was paused prior to vaccination, we screened for the development of *de novo* DSA or an increase of previously detected DSA. DSA of IgG isotype against mismatched donor HLA were determined by Luminex technology using the LABScreen Single Antigen kit of One Lambda, Inc. (West Hill, CA, United States). DSA with MFI ≥ 500 were considered positive as the incidence of graft loss has shown to be higher in patients with *de novo* DSA or non-DSA at an MFI ≥ 500 (49).

### Quantification of donor-derived cell-free DNA (dd-cfDNA)

dd-cfDNA constitutes a marker of graft injury and has been shown to significantly discriminate biopsy-confirmed rejection from no-rejection (50–53). Venous blood was drawn into 10 mL cell-free DNA BCT tubes (Streck, Omaha, NE, United States) and processed within 7 days. cfDNA was extracted using the Circulating Nucleic Acid kit (Qiagen, Redwood City, CA, United States) and amplified using the AlloSeq cfDNA assay (CareDx, Brisbane, CA, United States), a single multiplex PCR including index adapters and PCR primers for 202 single nucleotide polymorphisms (SNPs). Differences in SNPs loci are used to compute the amount of dd-cfDNA relative to the total amount of cfDNA from a sample. PCR products were sequenced on a MiSeq system (Illumina, San Diego, CA, United States). Data was analyzed using the AlloSeq cfDNA software (CareDx) which reports the percentage of donor-derived cfDNA. All steps were performed according to the manufacturers’ instructions. dd-cfDNA was measured in 40 patients before and in all 43 patients at 4 weeks follow-up after withdrawal of MPA.

### Reactogenicity

Reactogenicity after additional COVID-19 vaccination was assessed in all 76 KTR using a 12-item questionnaire to inquire about any adverse events following vaccination as described previously (Supplementary Methods) (38, 39, 46).

### Statistics

Data are given as median and interquartile range (IQR) or number (N) and percent (%). For continuous variables, the Mann–Whitney *U* test and the Wilcoxon matched-pairs rank test were applied for unpaired or paired variables, respectively. Fisher’s exact test was applied for categorial variables. A multiple linear regression analysis was performed to differentiate predictors of maximum anti-S1 IgG antibody levels in KTR with immunosuppressive maintenance therapy consisting of CNI, MPA, and CS. To describe the correlation of different commercially available assays to neutralization titers obtained by live-virus neutralization assays, we calculated Spearman’s rho as a non-parametric measure of rank correlation. Statistical analysis was performed using GraphPad Prism version 9.0.0 (GraphPad Software, San Diego, CA, United States) and statistical significance was assumed at a *P*-value < 0.05.

## Results

### Study population

We prospectively enrolled 76 KTR with no seroresponse after at least three prior COVID-19 vaccinations before administration of an additional dose with mRNA-1273 (100 μg). Five patients (7%) had four vaccinations, 71 patients (93%) 3 vaccinations prior to inclusion into the study. Median (IQR) age was 57 (47–63) years and 29/76 (38%) participants were females. Baseline characteristics including transplant-related data, cause of nephropathy and comorbidities are given in Table 1.

**TABLE 1:** Baseline characteristics.

All study participants, N	76
Age at enrollment (years), median (IQR)	57 (47–63)
Sex (female), N (%)	29 (38)
BMI (kg/m^2^), median (IQR)	24.8 (21.9–28.8)
**Vaccination related data^1^**	
Homologous mRNA vaccination, N (%)	51 (67)
Heterologous mRNA vaccination, N (%)	13 (17)
Heterologous vaccination including a viral vector	12 (16)
vaccine, N (%)	5 (7)
**More than three previous vaccine doses**	
Transplant-related data	
First transplant, N (%)	68 (89)
Time since transplantation (years), median (IQR)	4.7 (2.2–9.8)
Rejection during the past 12 months, N (%)	2 (3)
S-Creatinine prior to Vaccination (mg/dl)	1.5 (1.3–1.8)
S-Creatinine after Vaccination (mg/dl)	1.4 (1.2–1.7)
**Immunosuppressive maintenance therapy**	
CNI + MPA + CS, N (%)	68 (89)
Tacrolimus vs. Cyclosporine A, N (%)	51 (75) vs. 17 (25)
mTOR + CNI + CS, N (%)	4 (5)
mTOR + MPA + CS, N (%)	1 (1)
Belatacept + MPA + CS, N (%)	2 (3)
CNI + CS	1 (1)
**Cause of end-stage kidney disease**	
Vascular, N (%)	4 (5)
Diabetes, N (%)	7 (9)
Glomerular disease, N (%)	31 (41)
PKD, N (%)	15 (20)
Systemic, N (%)	2 (3)
Reflux/chronic pyelonephritis	6 (8)
Other/Unknown, N (%)	11 (14)
**Comorbidities**	
Arterial Hypertension, N (%)	57 (75)
Diabetes, N (%)	11 (14)
CAD, N (%)	18 (24)
Chronic lung disease, N (%)	11 (14)
Chronic liver disease, N (%)	5 (7)
Malignancy, N (%)	18 (24)

BMI, body-mass index; CAD, coronary artery disease; CNI, calcineurin inhibitor; CS, corticosteroids; MPA, mycophenolic acid; mTOR, mammalian target of rapamycin; N, number; PKD, polycystic kidney disease.

^1^Homologous mRNA vaccination: 49 KTR received three doses with BNT162b2; 2 KTR received four doses with BNT162b2; Heterologous mRNA vaccination: 5 KTR received two doses with BNT162b2 followed by one dose of mRNA-1273; 6 KTR received two doses of mRNA-1273 followed by one dose of BNT162b2; 2 received three doses of BNT162b2 followed by one dose of mRNA-1273; Heterologous vaccination including a viral vector vaccine: 7 received two doses of ChAdOx1 followed by one dose with BNT162b2; 1 received two doses of ChAdOx1 followed by one dose of mRNA-1273; 2 received one dose of ChAdOx1 followed by two doses of BNT162b2; 1 received one dose of ChAdOx1 followed by one dose of mRNA-1273 and one dose of BNT162b2; 1 received three doses with BNT162b followed by one dose of Janssen COVID-19 vaccine.

### Humoral immune responses in kidney transplant recipients as determined by commercially available assays

**FIGURE 2:**
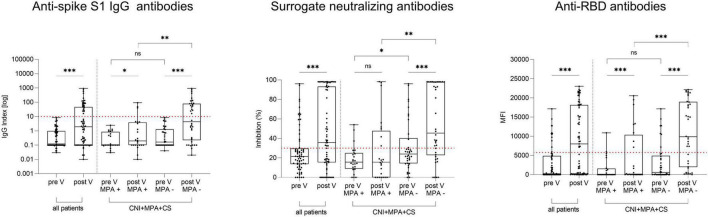
Anti-spike S1 IgG, surrogate neutralizing, and anti-receptor-binding domain antibodies in 69 kidney transplant recipients before and after an additional mRNA-1273 vaccine dose. Anti-spike S1 IgG (left panel), surrogate neutralizing (middle panel) and anti-RBD (right panel) antibodies in 69 KTR before and after additional COVID-19 vaccination. Results were stratified for 62 patients with triple immunosuppressive therapy consisting of a calcineurin inhibitor (CNI), mycophenolic acid (MPA), and corticosteroids (CS) according to temporary MPA withdrawal during vaccination (MPA + vs MPA -). KTR with breakthrough infections (*N* = 9) were excluded from all analyses. The dashed red line indicates the cut-off for detection of antibodies for each assay. CNI, calcineurin inhibitor; CS, corticosteroids; MFI, mean fluorescence intensity; MPA, mycophenolic acid; V, vaccination; ^***^P < 0.001; ^**^P < 0.01; *P < 0.05; ns, non-significant.

After vaccination, 24/69 (35%) KTR showed seroconversion with anti-spike S1 IgG antibodies above the predefined cut-off. Anti-spike S1 IgG index, % inhibition for surrogate neutralizing antibodies, and MFI for anti-RBD antibodies before vaccination increased from a median (IQR) of 0.12 (0.10–0.98) to 1.92 (0.10–47.18), from 21.6 (13.7–29.8) to 35.7 (15.4–93.2), and from 272 (0–4876) to 8,009 (206–18,149) after vaccination, respectively (*P* < 0.001 for all, Figure 2). When comparing KTR with immunosuppressive maintenance therapy consisting of CNI, MPA, and CS (*N* = 62) and stratifying for MPA withdrawal, 18/38 (47%) KTR in whom MPA was withdrawn showed seroconversion compared to 3/24 (13%) with continued immunosuppressive maintenance therapy including MPA (*P* = 0.006). Anti-S1 IgG index after vaccination was with a median (IQR) of 4.30 (0.22–78.8) significantly higher in patients with prior MPA withdrawal compared to the 0.20 (0.10–3.94) in those without MPA withdrawal (*P* = 0.006, Figure 2). Correspondingly, surrogate neutralizing and anti-RBD antibodies were significantly higher in patients where MPA was withdrawn compared to those without MPA withdrawal (*P* = 0.002 and *P* < 0.001, respectively, Figure 2). Patients with breakthrough infections (*N* = 7) were excluded from the analysis.

**FIGURE 3:**
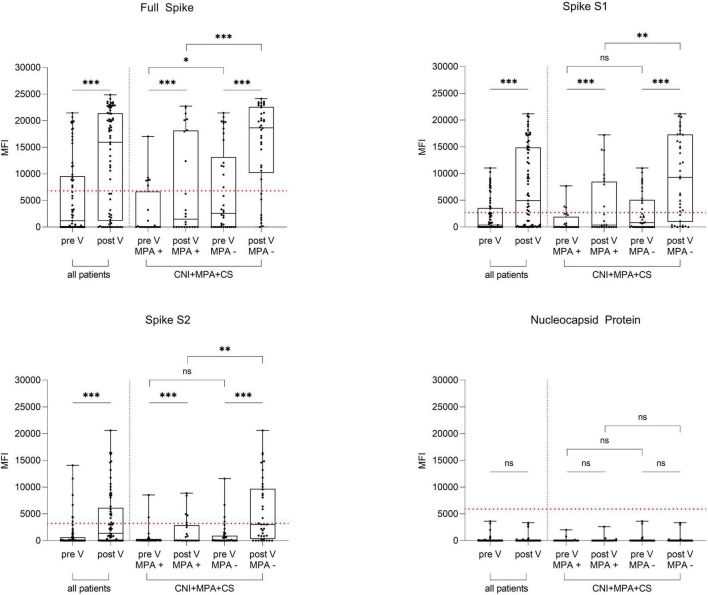
IgG antibodies against the full spike, the spike S1 and S2 subunits and the nucleocapsid protein in 69 kidney transplant recipients before and after an additional mRNA-1273 vaccine dose. IgG antibodies targeting the SARS-CoV-2 full spike (upper left panel), the spike S1 (upper right panel) and S2 subunits (lower left panel), and the nucleocapsid protein (lower right panel) were determined in 69 kidney transplant recipients (KTR) before and after additional vaccination using a multiplex bead-based assay. Results were stratified for 62 patients with triple immunosuppressive therapy consisting of a calcineurin inhibitor (CNI), mycophenolic acid (MPA), and corticosteroids (CS) according to temporary MPA withdrawal during vaccination (MPA + vs MPA -). KTR with breakthrough infections (*N* = 9) were excluded from all analyses. The dashed red line indicates the cut-off for each respective target. CNI, calcineurin inhibitor; CS, corticosteroids; KTR, kidney transplant recipients; MFI; mean fluorescence intensity; MPA, mycophenolic acid; V, vaccination; ^***^P < 0.001; ^**^P < 0.01; *P < 0.05; ns, non-significant.

In a multiplex bead-based assay, we determined antibodies targeting different areas of the spike protein (full spike, spike S1, spike S2) and antibodies targeting the nucleocapsid protein. In all KTR, spike-specific antibodies increased from 1,200 (0–9,558) to 15,921 (1,179–21,411), from 362 (0–3,547) to 4,948 (100–14,873), and from 0 (0–630) to 1,362 (0–6,126) for the full spike, the spike S1 and the spike S2 after additional vaccination, respectively (*P* < 0.001 for all, Figure 3). No significant differences in antibodies against the nucleocapsid protein were seen before and after vaccination (*P* = 0.46, Figure 3). When again stratifying results for patients where MPA was withdrawn prior to vaccination, antibodies against the full spike, the spike S1 and spike S2 subunits after additional vaccination were significantly higher in these patients compared to those who remained on maintenance therapy with MPA (*P* < 0.001 for antibodies against the full spike and the spike S1, *P* = 0.003 for antibodies against the spike S2, Figure 3). No significant differences were seen in antibodies against the nucleocapsid protein between the two groups after vaccination (*P* = 0.84, Figure 3). In addition, antibodies against 4 common cold coronaviruses were determined by this multiplex assay. We did not detect any significant differences in antibodies against the spike S1 of the HCoV-229E, the HCoV-HKU1, the HCoV-NL63, and the HCoV-OC43 before and after vaccination in all KTR and when stratified according to MPA withdrawal (Supplementary Figure 1).

In patients with maintenance immunosuppressive therapy consisting of CNI, MPA, and CS, a multiple linear regression analysis, including age, gender, time since transplantation, S-creatinine levels at time of vaccination, and MPA withdrawal upon vaccination was performed to identify possible confounders of maximum anti-S1 IgG levels (Supplementary Table 1). Besides MPA withdrawal (β: 100.7; 95% CI: 10.7; 190.7; *P* = 0.03), no other parameter examined was associated with higher anti-S1 IgG antibody concentrations (Supplementary Table 1). With the exception of a greater incidence of end-stage kidney disease caused by diabetes, no significant differences in baseline characteristics were detected when comparing KTR in whom MPA was paused during vaccination to those who remained on triple immunosuppressive maintenance therapy including MPA (Supplementary Table 2). KTR that underwent MPA withdrawal and did not seroconvert successfully were transplanted more recently than KTR with MPA withdrawal that showed seroconversion (*P* = 0.04; Supplementary Table 3).

### Neutralizing antibody response against the B.1.617.2 (delta) and B.1.1.529 (omicron) variants

**FIGURE 4:**
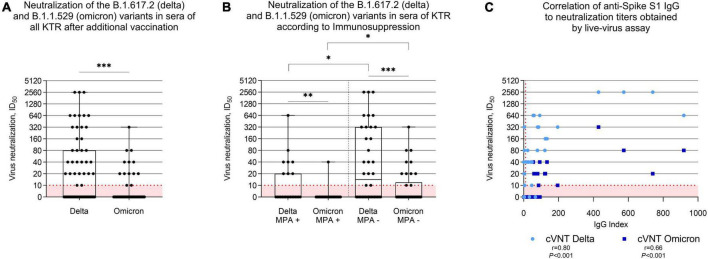
Neutralization of the SARS-CoV-2 B.1.617.2 (delta) and the B.1.1.529 (omicron) variants by antibodies in sera of 69 kidney transplant recipients after an additional mRNA-1273 vaccine dose. **(A)** Vaccine-induced cross-neutralization of the B.1.617.2 (delta) and the B.1.1.529 (omicron) variants by antibodies in sera of 69 kidney transplant recipients (KTR) after an additional mRNA-1273 vaccine dose as determined by using a live-virus assay. The dashed red line indicates the cut-off for detection which is the 1:10 dilution in this assay. **(B)** Cross-neutralization of the B.1.617.2 (delta) and the B.1.1.529 (omicron) variants by antibodies in sera of 62 KTR with maintenance immunosuppressive therapy consisting of a calcineurin inhibitor (CNI), mycophenolic acid (MPA), and corticosteroids (CS) stratified according to temporary MPA withdrawal during additional vaccination. **(C)** Correlation analysis of anti-S1 IgG results obtained by a commercially available assay with cross-neutralization titers of the B.1.617.2 (delta) and the B.1.1.529 (omicron) variants by sera of kidney transplant recipients taken after an additional mRNA-1273 vaccine dose. KTR with breakthrough infections (*N* = 9) were excluded from the analyses. cVNT, conventional virus neutralization test; ID_50_; inhibitory dilution 50; KTR, kidney transplant recipients; MPA, mycophenolic acid; r; Spearman’s rho; ^***^P < 0.001; ^**^P < 0.01; *P < 0.05.

Neutralization of the SARS-CoV-2 delta and omicron variants was determined with all KTR serum samples taken after vaccination using a live-virus assay. Neutralization titers were above 1:10 in 33/69 (48%) KTR for the delta variant, and in 13/69 (19%) KTR for the omicron variant. Neutralizing antibody titers for the delta variant were a median (IQR) ID_50_ of 0 (0–1:80) and significantly higher compared to the median (IQR) ID_50_ of 0 (0–0) for the omicron variant (*P* < 0.001, Figure 4A). When comparing patients where MPA was withdrawn prior to vaccination to those who remained on immunosuppressive maintenance therapy including MPA, the former exhibited significantly higher neutralization titers against both, the delta and omicron variant (*P* = 0.04 for delta and *P* = 0.02 for omicron, Figure 4B). A higher anti-S1 IgG antibody index correlated with higher neutralization titers of the delta and omicron variants (Figure 4C).

### Monitoring of patients with mycophenolic acid withdrawal

S-creatinine and proteinuria remained stable in KTR in whom MPA was withdrawn during vaccination with a median (IQR) S-creatinine of 1.4 mg/dl (1.3–1.8) and proteinuria of 18.7 g/molCrea (10.6–28.7) before vaccination compared to 1.4 mg/dl (1.3–1.6) and 19.6 g/molCrea (11.9–34.9) 4 weeks after vaccination, respectively (*P* = 0.5 and *P* = 0.13).

In addition, donor-specific anti-HLA antibodies (DSA) and donor-derived cell-free DNA were determined in all 43 patients with MPA withdrawal prior to and 4 weeks after withdrawal (Supplementary Table 4). In 29 patients, we did not detect any formation of *de novo* DSA. Two patients showed decreasing DSA reactivities below the cut-off of ≤ 500 during study period from a maximum MFI of 872 to 109 (DPB1*04:01) and 540–340 (C*07:02). In 7 patients we detected an increase of the MFI values of present DSA or a resurgence of previously detected DSA during MPA withdrawal. DSA could not be evaluated in two patients due to unavailable donor DNA for HLA typing and in three patients due to lacking of Luminex beads with the relevant HLA specificities. dd-cfDNA levels remained stable in all study participants with a median% of 0.14 (0.10–0.19) before and 0.14 (0.11–0.22) after MPA withdrawal (*P* = 0.11). In 42/43 (98%) KTR, dd-cfDNA remained below 0.5%, a cut-off that is strongly associated with likely risk for allograft injury (50, 54). dd-cfDNA only increased slightly in one patient from 0.51% to 0.65% without any corresponding changes in S-creatinine, proteinuria, or DSA levels (patient 32, Supplementary Table 4).

### Reactogenicity

Vaccination was overall well-tolerated in all KTR. Any side effect was reported by 43/76 (57%) KTR with local reactions being the most frequent reported in 35/76 (46%, Supplementary Figure 2). Side effects were distributed evenly in patients where MPA was withdrawn compared to those with continued maintenance immunosuppression including MPA with slightly more patients reporting use of medication in the group where MPA was withdrawn (Supplementary Figure 2).

## Discussion

In this study we found that a temporary halt of MPA prior to an additional COVID-19 vaccine booster enhanced seroconversion rates and lead to higher antibody levels for those KTR who had no prior seroresponse after at least three COVID-19 vaccinations. The reactogenicity profile was acceptable and showed mostly the typical expected local adverse events. After vaccination, 24/69 (35%) KTR showed seroconversion with anti-spike S1 IgG antibodies above the predefined cut-off. Correspondingly, KTR with higher anti-spike S1 IgG antibody levels exhibited higher levels of neutralizing antibodies targeting the B.1.617.2 (delta) and B.1.1.529 (omicron) variant. The 35% seroconversion rate we found is lower compared to other studies examining the effect of a fourth vaccine dose in KTR with seroconversion rates ranging between 42 and 50% (17–21). Lower seroconversion rates in our study cohort may be attributed to including only previous non-responder KTR in our trial. Of note, 18/38 (47%) KTR in whom MPA was paused surpassed the cut-off and showed significantly higher anti-spike S1 IgG antibodies compared to those who remained on triple immunosuppressive therapy. MPA withdrawal remained an independent variable associated with higher anti-spike S1 IgG antibodies when stratifying for age, gender, time since transplantation and S-Creatinine levels. In KTR with MPA withdrawal during vaccination we did not find any significant changes in S-creatinine, proteinuria or dd-cfDNA, indicative of no acute rejection (50–54). Although these findings may indicate immune quiescence, we detected resurgence in pre-existing DSA in 7 patients and the development of *de novo* DSA in one patient in whom MPA was withdrawn.

A few studies have examined the effect of MPA withdrawal to enhance vaccination responsiveness in small cohorts (55, 56). After showing a dose-dependent effect of MPA on antibody levels after two COVID-19 vaccinations, Kantauskaite et al. examined the effect of temporary MPA dose reduction by 25–50% in 24 KTR receiving a third mRNA vaccination matched to 24 KTR without changes in immunosuppressive maintenance therapy (31, 55). The authors found significantly higher antibody levels in patients with MPA reduction 3 weeks prior until 1 week after third vaccination, however, patients were not followed-up on graft function or development of DSA (55). Schrezenmeier et al. applied a fourth mRNA vaccine dose to 29 KTR during temporary halt of MPA and observed seroconversion in 76% of patients (56). Although the authors did not compare seroconversion rates to patients who remained on triple immunosuppressive therapy, their results are much in line with what we present in our current study. Higher seroconversion rates in their study cohort may apply to the fact that 52% in their study cohort received a heterologous vaccination protocol and median time since transplantation with 9.9 years (± SD 5.9) was longer than for our study cohort, both factors that have shown to influence seroconversion rates (57). Those KTR that failed to seroconvert despite MPA withdrawal in our study cohort were transplanted more recently compared to those that seroconverted successfully. This is consistent with present literature arguing that progressive dose reduction of immunosuppression with longer time since transplantation influences vaccine responsiveness in KTR (13, 33, 57, 58). Notably, Schrezenmeier et al. also followed-up on graft function, development of DSA and changes in dd-cfDNA and did not detect any differences when comparing pre-MPA withdrawal levels to post-MPA withdrawal levels (56). The resurgence of DSA in 7 of our patients may be attributed to the fact that we applied a lower cut-off (MFI ≤ 500), nevertheless we also detected HLA antibodies with MFI ≥ 1000 in 3 patients which is the cut-off Schrezenmeier et al. applied (56). Although no patient had a biopsy-confirmed rejection during study period, we think that MPA withdrawal in future trials may thus only be considered in patients without any prior DSA or current DSA to enhance safety.

Several studies showed reduced vaccine-elicited neutralization against omicron compared to SARS-CoV-2 wild-type even in healthy cohorts (59–62). Kumar et al. recently reported in a study cohort of 60 solid organ transplant recipients that only 55.0% and 18.3% of patients exhibited neutralizing antibody activity against delta and omicron 1 month after a third mRNA vaccine dose, respectively (28). In addition, first real-world data indicate a significantly reduced three-dose vaccine efficacy (95% CI) against infection with delta or omicron of 70.6% (31–87.5%) and 29.4% (0.3–50.0%) in immunocompromised individuals compared to 93.7% (92.2–94.9%) and 71.6% (69.7–73.4%) in the general population, respectively (63). Benotmane et al. recently showed in a cohort of 67 KTR with weak humoral responses after a third vaccine dose that 66% of patients were able to mount neutralizing antibodies against the delta variant after a fourth vaccine dose (21). Our results show even lower percentage of patients exhibiting neutralizing antibody activity against delta (48%) and omicron (19%) which again may be due to a selection bias only including non-responder KTR. After an additional, in most instances fourth mRNA vaccine dose in our study cohort of previous non-responder KTR, the 45/69 (65%) of patients that remained anti-spike S1 IgG seronegative and the concomitant reduced neutralization against the B.1.1.529 (omicron) variant remains distressing. This is in concordance with recently published results by Karaba et al. who reported that neutralization against the omicron variant did not increase significantly after additional vaccination in a cohort of 25 solid organ transplant recipients (SOTRs) with low seroresponse after three vaccinations, leaving SOTRs at high risk for omicron infection (64).

For KTR that fail to seroconvert even after adapted immunization protocols, pre-exposure prophylaxis with monoclonal antibodies remains an option although recent data suggests resistance of the newly surging BA.2 omicron sublineage to most available monoclonal antibodies (61, 65). The combination of Cilgavimab/Tixagevimab (Evusheld) has shown to retain partial neutralizing activity against the omicron variant *in vitro* and al Jurdi et al. recently demonstrated that SOTRs that received a pre-exposure prophylaxis with Evusheld at increased dosing of 300 mg of each antibody had significantly fewer breakthrough infections with omicron compared to SOTRs without pre-exposure prophylaxis and SOTRs that received the initially recommended dose of 150 mg of each antibody (66–68). As the COVID-19 pandemic continues to evolve and new and challenging variants of concern arise, the development of other safe and effective monoclonal antibodies that retain neutralization against the current SARS-CoV-2 variants remains a key aspect to safely protect immunocompromised patients who remain seronegative even after adapted immunization protocols.

There are several limitations to our study: this was a non-randomized single-center trial including 76 KTR with no vaccine response after at least three COVID-19 vaccinations. Larger, randomized multi-center trials and longer follow-up periods are needed to validate our results and evaluate clinical relevance and outcomes of MPA withdrawal before adapting vaccination protocols. Another limitation of our study is the lack of data on cellular immunity. Although neutralizing antibodies are seen as highly predictive of protection from symptomatic SARS-CoV-2 infection, our data do not fully reflect the immune response following COVID-19 vaccination (22). Further, although an increase in reactivities of DSA in some patients of our trial occurred during MPA withdrawal, we cannot eliminate the possibility that vaccination itself may have led to an alloimmune response. In this study, we aimed to investigate to what extent a reduction of immunosuppression (MPA withdrawal) is associated with an improved vaccination response without being associated with adverse events. Therefore, DSA and dd-cfDNA as early indicators of rejection were only measured in patients in whom MPA was withdrawn. In addition, serum MPA levels were not measured in either group to assess patient adherence, which could confound the results.

In conclusion, our data show a significant improvement in humoral immune response after an additional vaccine dose in previous non-responder KTR with at least three vaccine doses. Higher anti-S1 IgG antibody levels were associated with better neutralization of the B.1.617.2 (delta) and B.1.1.529 (omicron) variants. The effect was most pronounced in KTR where MPA was withdrawn 5–7 days prior to vaccination and remained paused for additional 4 weeks. Thus, MPA withdrawal or dose reduction seem reasonable approaches to enhance seroconversion rates. For safety reasons, this may be applied in patients without current or previous DSA.

## Data availability statement

The raw data supporting the conclusions of this article will be made available by the authors, without undue reservation.

## Ethics statement

The studies involving human participants were reviewed and approved by Ethics Committee of the University of Heidelberg. The patients/participants provided their written informed consent to participate in this study.

## Author contributions

LB and CSp analyzed and interpreted the data and drafted the manuscript. LB, TK, JB, MBu, CN, FK, MR, MT, MS, KK, and CSp collected and managed the data. LB, PS, CSü, TT, and CSp performed experiments on humoral response. MBa, HK, and RB performed experiments on live virus neutralization. CM, AB, PS, MZ, CSü, RB, and TT supervised the project and revised the manuscript. All authors critically reviewed the manuscript.
