# Supplementary material for: Humoral response to SARS-CoV-2 mRNA vaccination in previous non-responder kidney transplant recipients after short-term withdrawal of mycophenolic acid
Source: Front Med (Lausanne). 2022 Aug 18;9:958293. doi: 10.3389/fmed.2022.958293 (PMC9433830; doi:10.3389/fmed.2022.958293)
Supplement: Supplementary file 1 [file Data_Sheet_1.docx]

Supplementary Material

# Table of contents

Supplementary Table S1 Multiple linear regression analysis for baseline predictors of maximum anti-S1 IgG antibody concentration in 62 kidney transplant recipients with immunosuppressive maintenance therapy consisting of a calcineurin inhibitor, mycophenolic acid, and corticosteroids.

Supplementary Table S2 Baseline characteristics for 68 kidney transplant recipients with immunosuppressive maintenance therapy consisting of a calcineurin inhibitor, mycophenolic acid, and corticosteroids stratified for mycophenolic acid withdrawal.

Supplementary Table S3 Baseline characteristics for 38 kidney transplant recipients that underwent mycophenolic acid withdrawal prior to vaccination stratified for seroconversion defined as anti-spike S1 IgG antibody index > 10.

Supplementary Table S4 Serum-Creatinine, donor-specific anti-HLA antibody, and donor-derived cell-free DNA results in 43 kidney transplant recipients with mycophenolic acid withdrawal during vaccination.

Supplementary Figure S1 IgG antibodies against the spike S1 of four common cold coronaviruses in 69 kidney transplant recipients after an additional COVID-19 vaccination.

Supplementary Figure S2 Reactogenicity to additional COVID-19 vaccination in 76 kidney transplant recipients as assessed by a 12-item questionnaire.

Supplementary Methods

- Side effects questionnaire

Supplementary Table S1 Multiple linear regression analysis for baseline predictors of maximum anti-S1 IgG antibody concentration in 62 kidney transplant recipients with immunosuppressive maintenance therapy consisting of a calcineurin inhibitor, mycophenolic acid, and corticosteroids.

| **Characteristic** | **B** | **95% CI** | **SE** | ***p* Value** |
| --- | --- | --- | --- | --- |
| Age (years) | -2.7 | -6.5, 1.2 | 1.9 | 0.17 |
| Sex (female versus male) | -5.7 | -107.3, 95.9 | 50.7 | 0.91 |
| Time since TX | 2.4 | -6.1, 10.9 | 4.2 | 0.57 |
| S-Creatinine | 57.9 | -61.1, 177.0 | 59.4 | 0.33 |
| MPA withdrawal | 100.7 | 10.7, 190.7 | 44.9 | 0.03 (*) |

CI, confidence interval of regression coefficient B; B, regression coefficient; SE, standard error; TX, transplantation

Supplementary Table S2 Baseline characteristics for 68 kidney transplant recipients with immunosuppressive maintenance therapy consisting of a calcineurin inhibitor, mycophenolic acid, and corticosteroids stratified for mycophenolic acid withdrawal.

|  | **Maintenance IS with CNI+MPA+CS**  N = 68 | | ***P*-Value** |
| --- | --- | --- | --- |
|  | **Continued MPA therapy**  N = 25 | **MPA withdrawal**  N = 43 |  |
| Age at enrollment (years), median (IQR)  Sex (female), N (%)  BMI (kg/m^2^), median (IQR) | 56 (44–64)  11 (44)  24.1 (21.7–29.1) | 58 (50–63)  14 (33)  24.9 (21.6–28.6) | 0.46  0.44  0.93 |
| **Vaccination related data^1, 2^**  Homologous mRNA vaccination, N (%)  Heterologous mRNA vaccination, N (%)  Heterologous vaccination including a viral vector vaccine, N (%)  More than three previous vaccine doses | 19 (76)  3 (12)  3 (12)  1 (4) | 26 (60)  9 (21)  8 (19)  4 (9) | 0.29  0.51  0.73  0.64 |
| **Transplant-related data**  First transplant, N (%)  Time since transplantation (years), median (IQR)  Rejection during the past 12 months, N (%)  S-Creatinine before Vaccination (mg/dl)  S-Creatinine after Vaccination (mg/dl) | 21 (84)  3.7 (2.2–6.3)  1 (4)  1.6 (1.1–1.8)  1.6 (1.2–1.8) | 39 (91)  4.3 (2.0–9.3)  0 (0)  1.4 (1.3–1.8)  1.4 (1.3–1.6) | 0.45  0.69  /  0.74  0.57 |
| **Cause of end-stage kidney disease**  Vascular, N (%)  Diabetes, N (%)  Glomerular disease, N (%)  PKD, N (%)  Systemic, N (%)  Reflux/chronic pyelonephritis  Other/Unknown, N (%) | 2 (8)  6 (24)  6 (24)  4 (16)  1 (4)  1 (4)  5 (20) | 1 (2)  1 (2)  20 (47)  8 (19)  2(5)  4 (9)  7 (16) | 0.55  0.008  0.08  >0.99  >0.99  0.64  0.75 |
| **Comorbidities**  Arterial Hypertension, N (%)  Diabetes, N (%)  CAD, N (%)  Chronic lung disease, N (%)  Chronic liver disease, N (%)  Malignancy, N (%) | 18 (72)  4 (16)  6 (24)  2 (8)  4 (16)  3 (12) | 35 (81)  7 (16)  12 (28)  7 (16)  1 (2)  11 (26) | 0.38  >0.99  0.78  0.47  0.06  0.07 |

BMI, body-mass index; CAD, coronary artery disease; CNI, calcineurin inhibitor; CS, corticosteroids; MPA, mycophenolic acid; N, number; PKD, polycystic kidney disease

**^1^in continued MPA group**: Homologous mRNA vaccination: 18 KTR received three doses with BNT162b2; 1 KTR received four doses with BNT162b2; Heterologous mRNA vaccination: 1 KTR received two doses with BNT162b2 followed by one dose of mRNA-1273; 2 KTR received two doses of mRNA-1273 followed by one dose of BNT162b2; Heterologous vaccination including a viral vector vaccine: 3 received two doses of ChAdOx1 followed by one dose with BNT162b2

**^2^in MPA withdrawal group**: Homologous mRNA vaccination: 25 KTR received three doses with BNT162b2; 1 KTR received four doses with BNT162b2; Heterologous mRNA vaccination: 3 KTR received two doses with BNT162b2 followed by one dose of mRNA-1273; 4 KTR received two doses of mRNA-1273 followed by one dose of BNT162b2; 2 received three doses of BNT162b2 followed by one dose of mRNA-1273; Heterologous vaccination including a viral vector vaccine: 3 received two doses of ChAdOx1 followed by one dose with BNT162b2; 1 received two doses of ChAdOx1 followed by one dose of mRNA-1273; 2 received one dose of ChAdOx1 followed by two doses of BNT162b2; 1 received one dose of ChAdOx1 followed by one dose of mRNA-1273 and one dose of BNT162b2; 1 received three doses with BNT162b followed by one dose of Janssen COVID-19 vaccine

Supplementary Table S3 Baseline characteristics for 38 kidney transplant recipients that underwent mycophenolic acid withdrawal prior to vaccination stratified for seroconversion defined as anti-spike S1 IgG antibody index > 10.

|  | **MPA withdrawal**  N = 38 | | ***P*-Value** |
| --- | --- | --- | --- |
|  | **Seroconversion**  N = 18 | **No Seroconversion**  N = 20 |  |
| Age at enrollment (years), median (IQR)  Sex (female), N (%)  BMI (kg/m^2^), median (IQR) | 57 (43–61)  5 (28)  25.5 (21.8–28.7) | 60 (52–66)  7 (35)  24.8 (21.9–29.2) | 0.19  0.73  0.79 |
| **Vaccination related data**  Homologous mRNA vaccination, N (%)  Heterologous mRNA vaccination, N (%)  Heterologous vaccination including a viral vector vaccine, N (%)  More than three previous vaccine doses | 8 (44)  5 (28)  5 (28)  3 (17) | 14 (70)  3 (15)  3 (15)  1 (5) | 0.19  0.44  0.44  0.33 |
| **Transplant-related data**  First transplant, N (%)  Time since transplantation (years), median (IQR)  Rejection during the past 12 months, N (%)  S-Creatinine before Vaccination (mg/dl)  S-Creatinine after Vaccination (mg/dl) | 16 (89)  6.3 (3.1–10.3)  0 (0)  1.48 (1.23–1.78)  1.5 (1.28–1.63) | 18 (90)  2.4 (1.6–8.8)  0 (0)  1.41 (1.26–1.55)  1.41 (1.24–1.62) | >0.99  0.04  /  0.58  0.78 |
| **Cause of end-stage kidney disease**  Vascular, N (%)  Diabetes, N (%)  Glomerular disease, N (%)  PKD, N (%)  Systemic, N (%)  Reflux/chronic pyelonephritis  Other/Unknown, N (%) | 1 (6)  1 (6)  8 (44)  3 (17)  0 (0)  2 (11)  3 (17) | 0 (0)  0 (0)  10 (50)  4 (20)  1 (5)  1 (5)  4 (20) | /  /  0.76  >0.99  /  0.60  >0.99 |
| **Comorbidities**  Arterial Hypertension, N (%)  Diabetes, N (%)  CAD, N (%)  Chronic lung disease, N (%)  Chronic liver disease, N (%)  Malignancy, N (%) | 17 (94)  2 (11)  4 (22)  2 (11)  0 (0)  5 (28) | 16 (80)  5 (25)  7 (35)  5 (25)  0 (0)  5 (25) | 0.34  0.41  0.48  0.41  /  >0.99 |

BMI, body-mass index; CAD, coronary artery disease; MPA, mycophenolic acid; N, number; PKD, polycystic kidney disease

Supplementary Table S4 Serum-Creatinine, donor-specific anti-HLA antibody, and donor-derived cell-free DNA results in 43 kidney transplant recipients with mycophenolic acid withdrawal during vaccination.

|  | **Before MPA withdrawal** | | | | | **After MPA withdrawal** | | |
| --- | --- | --- | --- | --- | --- | --- | --- | --- |
| **Pat.** | **S-Creatinine [mg/dl]** | | **DSA**  **HLA specificities (MFI)** | | **dd-cfDNA [%]** | **S-Creatinine [mg/dl]** | **DSA**  **HLA specificities (MFI)** | **dd-cfDNA [%]** |
| 1 | 1,27 | | / | | 0,21 | 1,27 | / | 0,29 |
| 2 | 1,52 | | / | | 0,10 | 1,46 | / | 0,15 |
| 3 | 1,77 | | / | | n.a. | 1,6 | / | 0,49 |
| 4 | 1,04 | | / | | 0,15 | 1,32 | / | 0,34 |
| 5 | 1,38 | | / | | 0,08 | 1,24 | / | 0,09 |
| 6 | 1,37 | | / | | 0,27 | 1,41 | / | 0,21 |
| 7 | 1,79 | | C*01:02 (612) | | 0,14 | 1,44 | C*01:02 (759) | 0,08 |
| *Comment to patient 7: DSA testing 5 months prior to MPA withdrawal: C*01:02(560), DSA testing 2 months prior to MPA withdrawal: C*01:02(579)* | | | | | | | | |
| 8 | 0,86 | | / | | 0,26 | 0,9 | / | 0,28 |
| 9 | 1,52 | | / | | 0,17 | 1,58 | / | 0,15 |
| 10 | 1,71 | | / | | 0,07 | 1,76 | lacking of Luminex beads for  DRB1*08:04 | 0,09 |
| 11 | 1,42 | | / | | 0,08 | 1,31 | / | 0,20 |
| 12 | 1,56 | | / | | 0,23 | 1,64 | / | 0,47 |
| 13 | 1,96 | | / | | 0,16 | 2,02 | / | 0,14 |
| 14 | 1,28 | | / | | 0,11 | 1,36 | unavailable donor DNA for HLA typing | 0,21 |
| 15 | 0,89 | | / | | 0,12 | 0,89 | / | 0,11 |
| 16 | 1,26 | | C*07:02 (667) DRB5*01:01 (DR51) (213)  DPB1*04:01 (82) | | 0,1 | 1,23 | C*07:02 (809)  DRB5*01:01 (DR51) (3,009)  **DPB1*04:01(537)** | 0,1 |
| *Comment to patient 16: DSA testing 22 months prior to MPA withdrawal: DRB5*01:01 (DR51) (539), DSA testing 5 months prior to MPA withdrawal: C*07:02(634)* | | | | | | | | |
| 17 | 1,27 | | / | | 0,14 | 1,22 | / | 0,22 |
| 18 | 1,43 | | / | | 0,12 | 1,52 | / | 0,11 |
| 19 | 1,08 | | / | | 0,16 | 0,97 | / | 0,22 |
| 20 | 1,25 | | / | | 0,2 | 1,27 | / | 0,17 |
| 21 | 1,51 | | lacking of Luminex beads for B*18:14 | | 0,05 | 1,58 | lacking of Luminex beads for  B*18:14 | 0,07 |
| 22 | 1,32 | | DPB1*04:01 (872) | | 0,12 | 1,38 | DPB1*04:01 (109) | 0,11 |
| 23 | 1,97 | | / | | 0,18 | 1,71 | / | 0,14 |
| 24 | 1,28 | | DRB3*02:02 (DR52) (500) | | 0,05 | 1,38 | DRB3*02:02 (DR52) (943) | 0,05 |
| *Comment to patient 24: DSA testing 11 months prior to MPA withdrawal: DRB3*02:02(DR52) (458), DSA testing 5 months prior to MPA withdrawal: DRB3*02:02(DR52) (449)* | | | | | | | | |
| 25 | 1,83 | | / | | 0,11 | 1,56 | / | 0,12 |
| 26 | 1,19 | | / | | 0,19 | 1,11 | / | 0,22 |
| 27 | 1,47 | | / | | 0,21 | 1,4 | / | 0,14 |
| 28 | 1,75 | | / | | 0,24 | 1,64 | / | 0,29 |
| 29 | 1,88 | | A2 (1,631) | | 0,14 | 1,85 | A2 (2,590) | 0,13 |
| *Comment to patient 29: DSA testing 5 months prior to MPA withdrawal revealed A2 (4,368)* | | | | | | | | |
| 30 | 1,75 | | C*07:02 (540) | | 0,05 | 1,7 | C*07:02 (340) | 0,03 |
| 31 | 0,92 | | / | | 0,15 | 1,1 | / | 0,04 |
| 32 | 1,77 | | / | | 0,51 | 1,63 | / | 0,65 |
| 33 | 1,31 | | / | | 0,10 | 1,29 | / | 0,13 |
| 34 | 2,16 | | DQB1*02:02 (307) | | 0,14 | 1,94 | DQB1*02:02 (580) | 0,12 |
| *Comment to patient 34: DSA testing 6 months prior to MPA withdrawal: DQB1*02:02 (462)* | | | | | | | | |
| 35 | 1,62 | | / | | n.a. | 1,44 | / | 0,09 |
| 36 | 1,08 | | / | | 0,24 | 1,12 | / | 0,32 |
| 37 | 1,31 | | / | | 0,18 | 1,37 | / | 0,13 |
| 38 | 1,43 | | / | | 0,13 | 1,56 | / | 0,1 |
| 39 | 1,99 | | DRB4*01:01 (DR53) (476) | | 0,15 | 1,89 | DRB4*01:01 (DR53) (811) | 0,12 |
| *Comment to patient 39: DSA testing 60 months prior to MPA withdrawal: DRB4*01:01 (DR53) (689)* | | | | | | | | |
| 40 | 1,66 | | / | | 0,11 | 1,61 | / | 0,22 |
| 41 | 2,17 | | unavailable donor DNA for HLA typing | | 0,1 | 2,2 | unavailable donor DNA for HLA typing | 0,12 |
| 42 | 1,41 | | DQB1*06:03 (676) | | 0,18 | 1,58 | DQB1*06:03 (2,838) | 0,26 |
| *Comment to patient 42: DSA testing 19 months prior to MPA withdrawal:*  *DQB1*06:03 (377), DSA testing 14 months prior to MPA withdrawal: DQB1*06:03 (3,857)* | | | | | | | | |
| 43 | 1,58 | |  | | n.a. | 1,83 | lacking of Luminex beads for  DQA1*01:04 | 0,19 |
|  | | *De novo* DSA | |  |  |  |  |  |
|  | | *Increase* in prior DSA | |  |  |  |  |  |
|  | | *Decrease* in DSA | |  |  |  |  |  |

dd-cfDNA, donor-derived cell-free DNA; DSA, donor-specific anti-HLA antibodies; MPA, mycophenolic acid; n.a., not available; pat, patient

Supplementary Figure S1 IgG antibodies against the spike S1 of four common cold coronaviruses in 69 kidney transplant recipients after an additional COVID-19 vaccination.


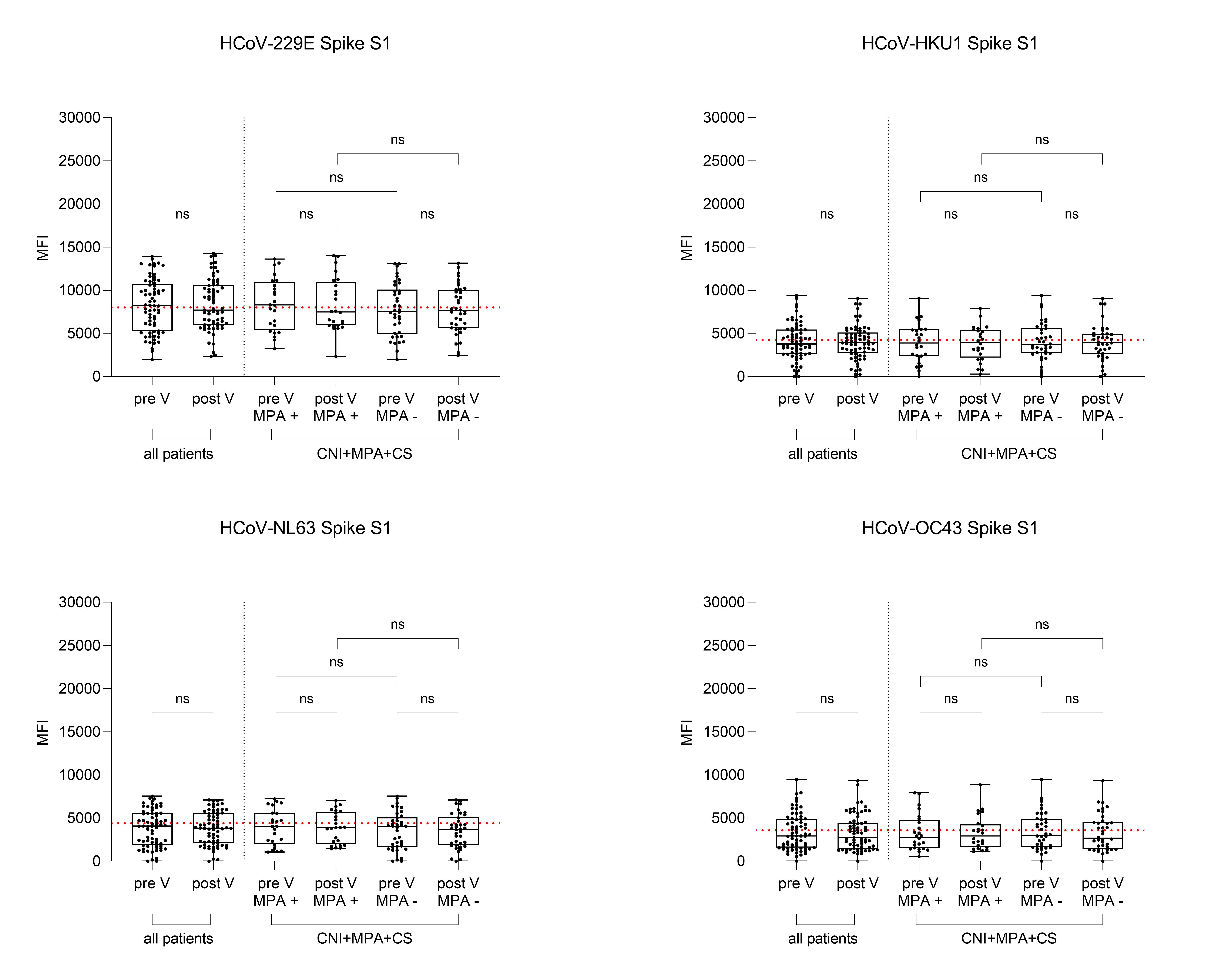


IgG antibodies against the spike S1 of four common cold coronaviruses, namely the HCoV-220E, HCoV-HKU1, HCoV-NL63, and HCoV-OC43 in 69 kidney transplant recipients before and after an additional vaccine dose. Results are stratified for 62 KTR with maintenance immunosuppressive therapy consisting of a calcineurin inhibitor, mycophenolic acid, and corticosteroids stratified according to continued mycophenolic acid (MPA +) and temporarily paused MPA (MPA -). The y-axis represents the mean fluorescence intensity (MFI), and the dashed red line indicates the cut-off for each target. KTR, kidney transplant recipients, MFI, mean fluorescence intensity; MPA, mycophenolic acid; V, vaccination; ns, non-significant.

Supplementary Figure S2 Reactogenicity to additional COVID-19 vaccination in 76 kidney transplant recipients as assessed by a 12-item questionnaire.


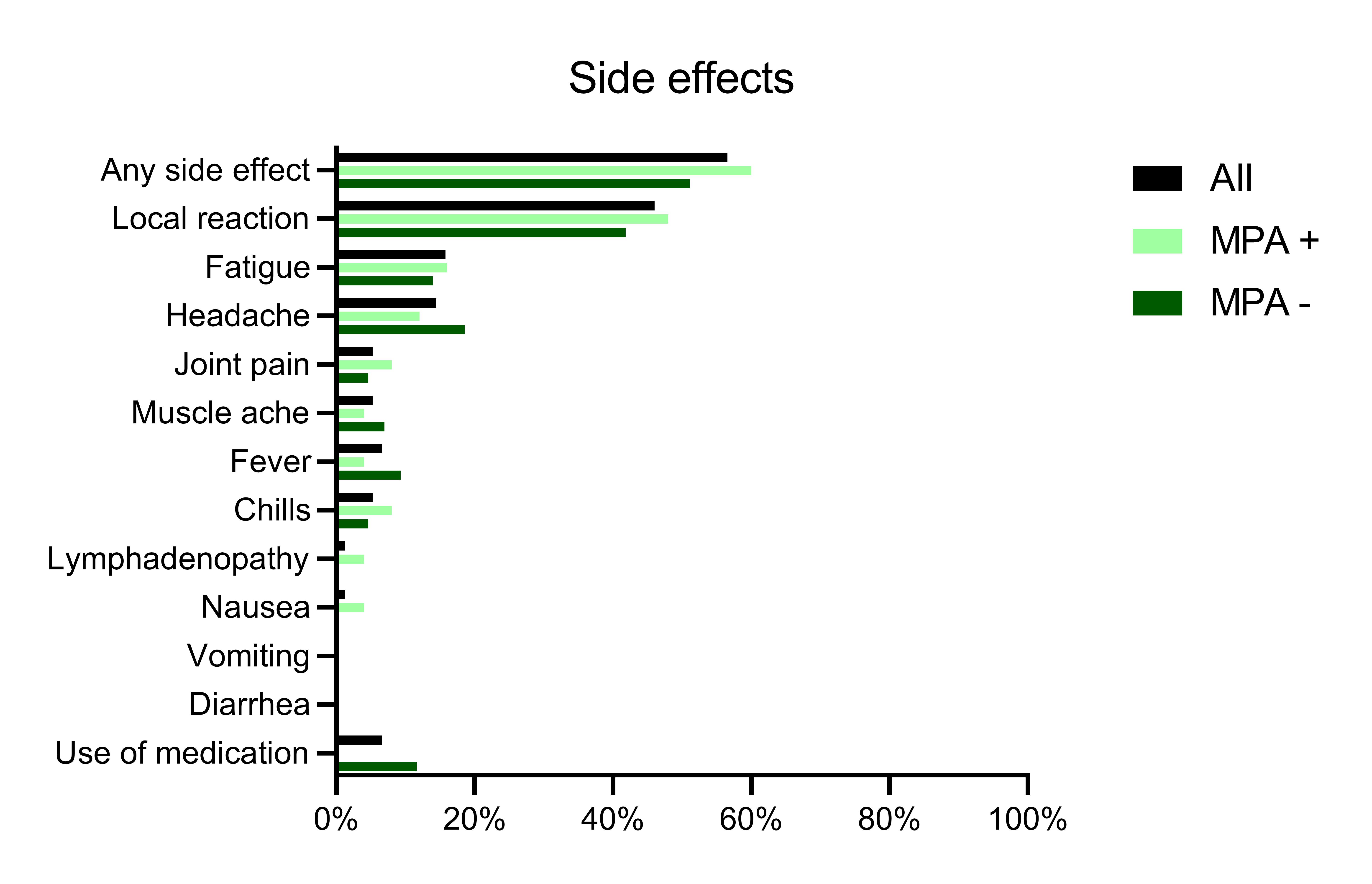


Reactogenicity was assessed for all 76 kidney transplant recipients receiving additional vaccination using a 12-item questionnaire inquiring about any reaction to vaccination, local reaction (including redness, swelling, pain), fatigue, headache, joint pain, muscle ache, fever, chills, lymphadenopathy, nausea, vomiting, diarrhea, and the use of medication. Results were analyzed separately in those that remained on triple immunosuppressive therapy including mycophenolic acid (MPA +) and those were mycophenolic acid was temporarily paused during vaccination (MPA -).

**Supplementary Methods**

Side effects questionnaire

| **Side effects questionnaire** | | | |
| --- | --- | --- | --- |
|  | After first vaccination | After second vaccination | After third vaccination |
| **Any** (yes/no) |  |  |  |
| If **yes**, please mark the appropriate side effects you had: | | | |
|  | After first vaccination | After second vaccination | After third vaccination |
| Local reaction (such as pain at the injection site, redness, swelling) |  |  |  |
| Fatigue |  |  |  |
| Headache |  |  |  |
| Joint pain |  |  |  |
| Muscle ache |  |  |  |
| Fever (≥38°C) |  |  |  |
| Chills |  |  |  |
| Lymphadenopathy |  |  |  |
| Nausea |  |  |  |
| Vomiting |  |  |  |
| Diarrhea |  |  |  |
| Use of medication |  |  |  |
